# *WOX11*: the founder of plant organ regeneration

**DOI:** 10.1186/s13619-022-00140-9

**Published:** 2023-01-04

**Authors:** Qihui Wan, Ning Zhai, Dixiang Xie, Wu Liu, Lin Xu

**Affiliations:** 1 National Key Laboratory of Plant Molecular Genetics, CAS Center for Excellence in Molecular Plant Sciences, Institute of Plant Physiology and Ecology, Chinese Academy of Sciences, 300 Fenglin Road, Shanghai, 200032 China; 2 University of Chinese Academy of Sciences, 19A Yuquan Road, Beijing, 100049 China

**Keywords:** Plant regeneration, De novo root regeneration, Callus, Adventitious lateral root, Adventitious root, *WOX11*

## Abstract

*De novo* organ regeneration is the process in which adventitious roots or shoots regenerate from detached or wounded organs. *De novo* organ regeneration can occur either in natural conditions, e.g. adventitious root regeneration from the wounded sites of detached leaves or stems, or in in-vitro tissue culture, e.g. organ regeneration from callus. In this review, we summarize recent advances in research on the molecular mechanism of *de novo* organ regeneration, focusing on the role of the *WUSCHEL-RELATED HOMEOBOX11* (*WOX11*) gene in the model plant *Arabidopsis thaliana*. *WOX11* is a direct target of the auxin signaling pathway, and it is expressed in, and regulates the establishment of, the founder cell during *de novo* root regeneration and callus formation. *WOX11* activates the expression of its target genes to initiate root and callus primordia. Therefore, *WOX11* links upstream auxin signaling to downstream cell fate transition during regeneration. We also discuss the role of *WOX11* in diverse species and its evolution in plants.

## Background

*De novo* organ regeneration is a type of plant regeneration, and refers to the ability of detached or wounded organs to regenerate adventitious roots and/or adventitious shoots (Ikeuchi et al. 2019; Sang et al. 2018; Williams 2021; Xu and Huang 2014). *De novo* organ regeneration can occur in natural conditions, for example, roots or shoots can regenerate from wounded leaves or stems. This process of direct *de novo* organ regeneration is widely exploited in the use of leaf or stem cuttings to propagate plants (Druege et al. 2019, 2016). *De novo* organ regeneration can also occur indirectly in tissue culture, in which adventitious roots or adventitious shoots are regenerated from callus, a pluripotent cell mass induced from detached explants by a high concentration of auxin in the medium (Ikeuchi et al. 2013; Sang et al. 2018; Sugimoto et al. 2011). Research on *de novo* organ regeneration has identified *WUSCHEL-RELATED HOMEOBOX11* (*WOX11*) as the key gene involved in the auxin response and cell fate transition. In this review, we mainly focus on the role of *WOX11* in the model plant *Arabidopsis thaliana*, including its role in *de novo* root regeneration from detached leaves, regeneration of adventitious lateral roots from the wounded primary root, and callus formation in tissue culture. We also summarize the conserved role of *WOX11* in diverse plant species and propose its evolutionary route in vascular plants.

## Role of *WOX11* in *de novo* root regeneration from detached leaves

Cutting technology is widely used for vegetative propagation of plants. Detached cuttings of leaves or stems regenerate adventitious roots from the wounded site, in a process known as *de novo* root regeneration (Bellini et al. 2014; Bustillo-Avendaño et al. 2018; Druege et al. 2019; De Klerk et al. 1999; Verstraeten et al. 2014; Xu 2018). Studies focusing on adventitious rooting from detached leaves of *Arabidopsis* (i.e., leaf cuttings) have revealed the developmental framework of *de novo* root regeneration, which can be separated into three phases (Xu 2018). In phase I, the detached leaf senses many signals including wounding and environmental signals as well as its own developmental status, and then converts all these signals to biosynthesize a certain level of auxin as the output in mesophyll, leaf margin, and some vasculature cells (together known as converter cells) (Chen et al. 2016a; Chen et al. 2016b; Hernández-Coronado et al. 2022; Li et al. 2020a; Pan et al. 2019; Shanmukhan et al. 2021; Ye et al. 2020; Zhang et al. 2019). The auxin level might be one of the factors determining the efficiency of root regeneration. In phase II, auxin is transported from the converter cells to the regeneration-competent cells (i.e. vascular adult stem cells such as procambium and some vascular parenchyma cells in the vasculature near the wounded site) (Chen et al. 2016a; Liu et al. 2014b; Sun et al. 2016). In phase III, the regeneration-competent cells undergo cell fate transition and division to form the root tip, guided by auxin (Bustillo-Avendaño et al. 2018; Hu and Xu 2016; Liu et al. 2014b; Liu et al. 2022; Shanmukhan et al. 2021; Sheng et al. 2017) Therefore, auxin is the key hormone in *de novo* root regeneration from detached *Arabidopsis* leaves.

**Fig. 1:**
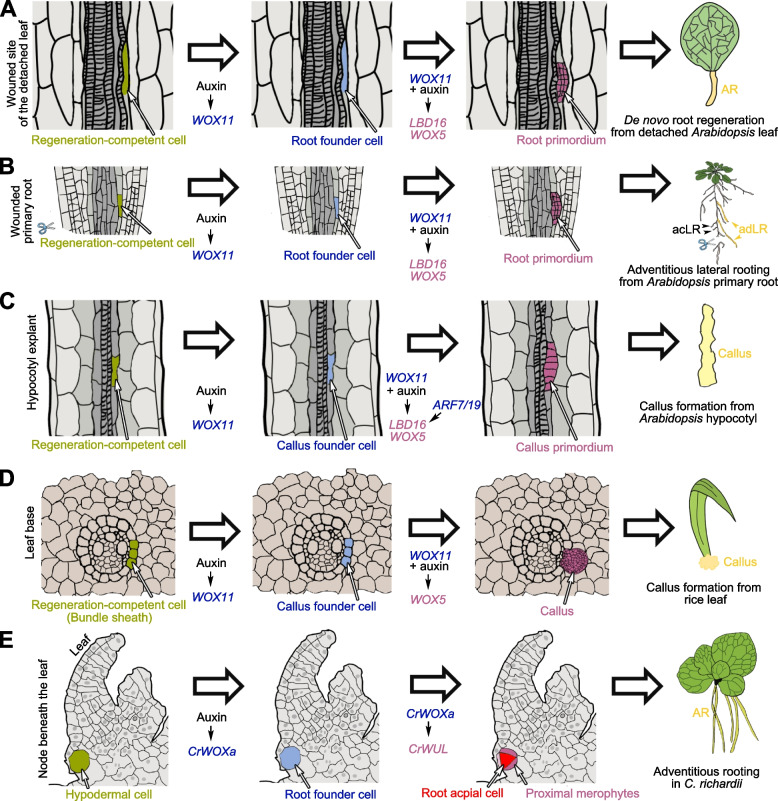
Roles of *WOX11* in *Arabidopsis* and rice, and *CrWOXa* in *Ceratopteris richardii*. **A** Role of *WOX11* in *de novo* root regeneration from detached *Arabidopsis* leaves. **B** Role of *WOX11* in adventitious lateral rooting from primary roots of *Arabidopsis*. **C** Role of *WOX11* in callus formation from *Arabidopsis* hypocotyl explants. **D** Role of *WOX11* in callus formation from rice leaf explants. **E** Role of *CrWOXa* in adventitious root initiation in *C. richardii*

*WOX11* was discovered in both *Arabidopsis* and rice (*Oryza sativa*) for its genetic role in promotion of adventitious rooting (Liu et al. 2014b; Zhao et al. 2009). During *de novo* root regeneration from detached *Arabidopsis* leaves, *WOX11* links auxin to the cell fate transition of regeneration-competent cells. The promoter region of *WOX11* harbors auxin response elements (AuxREs) that are targeted by AUXIN RESPONSE FACTORs (ARFs) in the auxin signaling pathway (Liu et al. 2014b). When auxin is polarly transported into the regeneration-competent cells, the auxin signaling pathway can directly induce *WOX11* expression via these AuxREs in regeneration-competent cells (Liu et al. 2014b), although it is still unclear which specific ARFs are involved in this process. The expression of *WOX11* indicates the cell fate transition from a regeneration-competent cell into an adventitious root founder cell (known as the priming step), and *WOX11* is the specific marker of root founder cells (Liu et al. 2014b). This cell fate transition process is not dependent on cell division. Next, as a transcription factor, WOX11 can directly bind to the WOX-binding *cis* elements (WOXCEs) in the promoters of *LATERAL ORGAN BOUNDARIES DOMAIN16* (*LBD16*), *WOX5*, and *WOX7*, and activate their expression (Hu and Xu 2016; Liu et al. 2014b; Sheng et al. 2017). Meanwhile, the adventitious root founder cell repeatedly divides to form the adventitious root primordium (known as the initiation step). *WOX11* expression decreases during cell division and *LBD16* and *WOX5*/*7* maintain their expression in the adventitious root primordium as molecular markers (Hu and Xu 2016; Liu et al. 2014b; Sheng et al. 2017). Differentiation of the adventitious root primordium leads to the formation of the adventitious root apical meristem (RAM, known as the patterning step) and finally to the formation of the adventitious root tip (known as the emergence step) (Xu 2018). Genetic studies indicate that mutation of *WOX11* or inhibition of the *WOX11* pathway can lead to decreased rooting ability, and overexpression of *WOX11* can increase the ability of detached leaves to form roots (Liu et al. 2014b; Pan et al. 2019). In addition, *WOX12* plays a partially redundant role with *WOX11* in *de novo* root regeneration from detached leaves (Liu et al. 2014b; Pan et al. 2019). *WOX11* and *WOX12* (*WOX11*/*12*) might act with *ARABIDOPSIS TRITHORAX-RELATED 2* (*ATXR2*), which is involved in epigenetic regulation of gene expression, during *de novo* root regeneration from detached *Arabidopsis* leaves (Lee et al. 2018a). Overall, *WOX11* promotes the cell fate transition of the regeneration-competent cell guided by auxin and participates in the priming and initiation steps (Fig. 1A).

In many other plant species, *WOX11* promotes *de novo* root regeneration as well as adventitious root formation in non-regeneration processes. In the *Populus* genus, *WOX11* can promote *de novo* root regeneration from stem cuttings, and overexpression of *WOX11* can significantly enhance adventitious rooting from detached stems (Bannoud and Bellini 2021; Li et al. 2018; Liu et al. 2014a; Liu et al. 2018b; Wang et al. 2019; Xu et al. 2015). In rice, *WOX11* is involved in the formation of crown roots, a type of adventitious root in monocots, probably through cooperation with epigenetic pathways (Chen et al. 2013; Cheng et al. 2018; Jiang et al. 2017; Li et al. 2017; Lu et al. 2012; Mao et al. 2020a; Panda et al. 2021; Zhang et al. 2018b; Zhao et al. 2009, 2020, 2015; Zhou et al. 2017). *WOX11* also regulates adventitious rooting in *Panax ginseng* (Liu et al. 2019), the banyan tree (*Ficus macrocarpa*) (Zhang et al. 2020), and apple (*Malus domestica*) (Mao et al. 2020b). Overall, it seems that *WOX11* has at least a partially evolutionarily conserved role in promoting adventitious rooting in angiosperms, and could be a useful molecular tool to promote rooting from leaf or stem cuttings.

## Role of *WOX11* in the formation of adventitious lateral roots from wounded roots

In seed plants, the root system architecture is usually established by three types of roots: the primary root, which is the first root formed in the embryo stage; lateral roots, which initiate from developing roots; and adventitious roots, which initiate from non-root organs or very old roots (Barlow. 1994, 1986; Groff and Kaplan 1988; Rost et al. 1997). Two types of lateral roots contribute to the plasticity of root system architecture in many plant species: acropetal lateral roots, which initiate from the tip of a developing parent root and emerge in an acropetal sequence; and adventitious lateral roots, which initiate without the acropetal pattern and can continuously form between acropetal lateral roots or even during secondary growth of the parent root (Barlow 1986; Charlton 1996; Esau 1965; Ge et al. 2019; Hou et al. 2004; Paolillo and Zobel 2002; Priestley and Swingle 1929).

In wild-type *Arabidopsis* growing vertically on synthetic medium, the primary root usually produces acropetal lateral roots. During acropetal lateral root initiation, ARF7/19 in the auxin signaling pathway directly activate *LBD16* and other *LBD* genes in the acropetal lateral root founder cells to initiate the acropetal lateral root primordium (Ito et al. 2016; Okushima et al. 2007, 2005). Mutations in *ARF7*/*19* can result in loss of *LBD*s expression and the lack of acropetal lateral root formation from the primary root in *Arabidopsis* (Okushima et al. 2007, 2005). *WOX11* is not expressed in the acropetal lateral root founder cells and is not required for the initiation of the acropetal lateral root primordium (Liu et al. 2014b; Sheng et al. 2017). However, when the primary root is cut or under severe stress, adventitious lateral roots are able to regenerate from the primary root, even in the *arf7 arf19* double mutant background (Ditengou et al. 2008; Sheng et al. 2017). *WOX11* is expressed in the adventitious lateral root founder cell and directly activates *LBD16* expression to initiate the adventitious lateral root primordium independently of *ARF7*/*19* (Sheng et al. 2017). Because auxin is also critical for adventitious lateral rooting, it is expected that *ARF*s other than *ARF7*/*19* might cooperate with *WOX11* in this process. Interestingly, both acropetal and adventitious lateral roots contribute to root system architecture when wild-type *Arabidopsis* is grown in soil. In soil, *WOX11* also contributes to adventitious lateral rooting, thereby contributing to the formation of normal root system architecture (Sheng et al. 2017). In addition, *WOX11* is involved in the formation of adventitious lateral roots from secondary growth of the primary root in *Arabidopsis* (Baesso et al. 2018). Thus, similar to its role in *de novo* root regeneration from detached *Arabidopsis* leaves, *WOX11* also functions in the root founder cells during adventitious lateral root formation (Fig. 1B).

The involvement of *WOX11* in adventitious lateral rooting has been reported for many other species. In *Populus*, application of a bending treatment to the woody taproot with secondary structures causes adventitious lateral roots to initiate from the vascular cambium zone, and *WOX11* expression is highly induced during this process (Baesso et al. 2020). In *Malus hupehensis*, indole-3-butanoic acid (IBA) treatment induces adventitious lateral rooting, probably via the *WOX11* pathway (Mao et al. 2018). In radish (*Raphanus sativus*), *WOX11*-mediated adventitious lateral roots initiate from the cambium of the storage taproot upon wounding (Aliaga Fandino et al. 2019). In rice, there are two types of lateral roots: S-type lateral roots that are short and thin and lose their ability to produce higher-order lateral roots; and L-type lateral roots that are long and thick and are able to further produce lateral roots. Wild-type rice predominantly produces S-type lateral roots from the primary root, while the L-type lateral roots are induced upon root tip cutting or stress treatment of the primary root via a process involving *WOX11* in addition to other *WOX* genes (Kawai et al. 2022). It will be interesting to further investigate whether the initiation of S-type lateral roots in rice is similar to that of the acropetal lateral root in *Arabidopsis* and does not require *WOX11* (Zhao et al. 2009; Zhu et al. 2012), and whether the L-type lateral roots are similar to the *WOX11*-mediated adventitious lateral roots in *Arabidopsis*. In addition, *WOX11* might be an efficient molecular tool for the improvement of the root system in response to diverse stress soil conditions in rice (Chen et al. 2015; Cheng et al. 2016) and *Populus* (Wang et al. 2019). Overall, the *WOX11*-mediated adventitious lateral rooting pathway might contribute to the plasticity of root system formation in many plant species.

## Role of *WOX11* in callus formation

Tissue culture is a widely used plant biotechnology for vegetative propagation (Ikeuchi et al. 2019; Skoog and Miller 1957). Usually, at least two types of callus can form during tissue culture of explants, i.e., embryonic callus and shooty/rooty callus (Ikeuchi et al. 2013). Here, we summarize the roles of *WOX11* in shooty/rooty callus (hereafter referred to as callus). In *Arabidopsis*, callus initiates from detached explants in response to a high-auxin-to-low-cytokinin ratio on callus-inducing medium (CIM). The regeneration-competent cells that initiate callus are those that are able to initiate lateral or adventitious roots, i.e. vascular adult stem cells including xylem-pole pericycle, procambium, and some vascular parenchyma cells (Atta et al. 2009; Che et al. 2007; Hu et al. 2017; Liu et al. 2014b; Sugimoto et al. 2011, 2010). Callus is able to regenerate shoots in response to a high-cytokinin-to-low-auxin ratio on shoot-inducing medium (SIM) (Cheng et al. 2013; Dai et al. 2017; Gordon et al. 2007; Iwase et al. 2017; Kareem et al. 2015; Meng et al. 2017; Zhang et al. 2017), or to regenerate roots in response to a low auxin concentration on root-inducing medium (RIM) (Yu et al. 2017). Therefore, callus is a group of pluripotent cells that is competent for *de novo* organ regeneration, i.e., either root regeneration or shoot regeneration. The formation of callus in *Arabidopsis* borrows the lateral or adventitious root organogenesis pathway in plants (Duclercq et al. 2011; Fan et al. 2012; He et al. 2012; Liu et al. 2014b; Sugimoto et al. 2011, 2010), and the cellular structure of callus on CIM resembles that of the root primordium or the root apical meristem (Hu et al. 2017; Motte et al. 2014; Sugimoto et al. 2010; Zhai and Xu 2021). However, cell division in the root primordium or the root apical meristem is strictly and developmentally controlled, while cell division is more extensive in callus and is stimulated by a high concentration of exogeneous auxin.

In *Arabidopsis,* the cell fate transition during callus initiation is similar to that during adventitious root initiation (Fig. 1C). In callus forming on CIM, auxin promotes the expression of *WOX11* during the cell fate transition from regeneration-competent cells to callus founder cells (Hu et al. 2017; Liu et al. 2014b). Then WOX11, together with auxin, activates *LBD16* and *WOX5*/*7* expression during the division of callus founder cells to form the callus primordium (Hu and Xu 2016; Liu et al. 2018a, Liu et al. 2014b; Sheng et al. 2017). *LBD16* could alternatively be activated by the lateral rooting pathway involving the calcium (Ca^2+^) signaling module CALMODULIN IQ-MOTIF CONTAINING PROTEIN (CaM–IQM), *INDOLE-3-ACETIC ACID INDUCIBLE14* (*IAA14*) and *19*, and *ARF7* and *19* (Fan et al. 2012; Shang et al. 2016; Zhang et al. 2022). Besides *LBD16* and *WOX5*/*7*, many root stem cell-related genes are also highly induced during the formation of the callus primordium, including *PLETHORA1* and *2* (*PLT1*/*2*) and *SCARECROW* (*SCR*) (Fan et al. 2012; Gordon et al. 2007; Hu et al. 2017; Kareem et al. 2015; Kim et al. 2018; Liu et al. 2018a, Liu et al. 2014b; Sugimoto et al. 2010; Zhai and Xu 2021). *PLT3*/*5*/*7* are expressed during all stages of callus formation (Kareem et al. 2015). The loss or inhibition of the above key genes may result in the loss of pluripotency in callus, leading to shoot and/or root regeneration defects (Fan et al. 2012; Gordon et al. 2007; Hu et al. 2017; Hu and Xu 2016; Kareem et al. 2015; Kim et al. 2018; Liu et al. 2018a, Liu et al. 2014b; Sheng et al. 2017; Sugimoto et al. 2010; Zhai and Xu 2021). After initiation, the callus primordium continues to undergo cell division and partial differentiation with patterning of tissues to form the mature callus. Our recent study using single-cell RNA sequencing indicates that mature callus has at least three cell layers: the outer cell layer, which resembles the epidermis and lateral root cap of a root tip; the middle cell layer, which has quiescent center (QC)-like identity and is the pluripotent cell layer responsible for further organ regeneration; and the inner cell layer, which is similar to the vascular initial cells of the RAM (Zhai and Xu 2021). Therefore, the pluripotent cells for organ regeneration could be governed by the QC-like identity and are predominantly maintained in the middle cell layer (Zhai and Xu 2021). Additionally, the formation of callus, its acquisition of pluripotency, and subsequent organ regeneration require the cooperation of the epigenetic network that regulates the expression of the above key genes (He et al. 2012; Ishihara et al. 2019; Kim et al. 2018; Lee et al. 2021, 2019, 2018b; Wu et al. 2022; Zhao et al. 2020).

Among the above-ground tissues of rice, only the immature region of the leaf (the leaf base) and the node of the stem are able to form callus; it cannot initiate from the mature region of the leaf or the internode of the stem, because the vascular adult stem cells are fully differentiated into functional tissues and are not maintained during the maturation of above-ground organs (Hu et al. 2017). This differs from the *Arabidopsis* mature leaf, which retains vascular adult stem cells and the ability to initiate callus throughout its whole life (Hu et al. 2017). In rice, callus can initiate from the immature bundle sheath at the leaf base, and from the phloem-pole pericycle cells in the root (Hu et al. 2017). The phloem-pole pericycle cells are also responsible for lateral root initiation in rice. The cell fate transition from regeneration-competent cells (i.e. immature bundle sheath and phloem-pole pericycle) to callus founder cells in rice also requires *WOX11*, and *WOX5* is highly expressed in the callus primordium and mature callus, similar to the situation during callus initiation in *Arabidopsis* (Hu et al. 2017) (Fig. 1D). In addition to *WOX11*, the *OsIAA11*-mediated lateral root initiation pathway contributes to callus initiation in rice roots (Guo et al. 2018). Overall, the molecular pathway for callus initiation in rice and *Arabidopsis* share many similar molecular modules, while the tissues/cells that are able to initiate callus are dependent on the species-specific developmental program.

## Evolutionary route of *WOX* genes

**Fig. 2:**
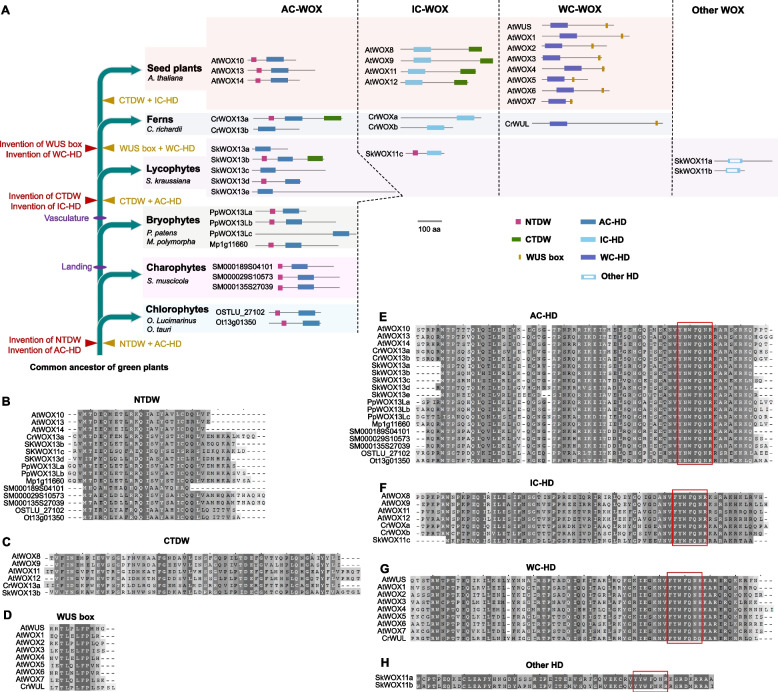
Domain evolution of WOX family proteins. **A** Evolution of green plants, showing predicted emergence of domains and combination of domains (indicated by +) in WOX family proteins. WOX proteins and their domains in *Ostreococcus lucimarinus*, *Ostreococcus tauri*, *Spirogloea muscicola*, *Physcomitrella patens*, *Marchantia polymorpha*, *Selaginella kraussiana*, *Ceratopteris richardii*, and *Arabidopsis thaliana* are shown. **B–H** Alignment of NTDW (**B**), CTDW (**C**), WUS box (**D**), AC-HD (**E**), IC-HD (**F**), WC-HD (**G**), and other HD (**H**). Red boxes indicate the specific sequences in HD

The WOX family genes in *Arabidopsis* can be grouped into three clades on the basis of their encoded domains and motifs, i.e. the ancient-clade *WOX* (AC-*WOX*) genes, the intermediate-clade *WOX* (IC-*WOX*) genes, and the WUS-clade *WOX* (WC-*WOX*) genes (Ge et al. 2016; van der Graaff et al. 2009; Haecker 2004; Nardmann and Werr 2012; Zhang et al. 2010) (Fig. 2A-G). *Arabidopsis* AC-*WOX* genes, including *WOX10*, *WOX13,* and *WOX14*, encode proteins with the typical N-terminal domain of WOX (NTDW) and the AC-type homeodomain (AC-HD) with the typical YNWFQNR sequence (Fig. 2A, B, E). *Arabidopsis* IC-*WOX* genes, including *WOX11*, *WOX12*, *WOX8,* and *WOX9*, encode proteins with a typical C-terminal domain of WOX (CTDW) and the IC-type homeodomain (IC-HD) with the typical FYWFQNR sequence (Fig. 2A, C, F). *Arabidopsis* WC-*WOX* genes, including *WUS* and *WOX1* to *WOX7*, encode proteins with the typical WUS box and the WC-type homeodomain (WC-HD) with the typical FYWFQNH sequence (Fig. 2A, D, G). *WOX* genes are present in almost all green plants from algae to seed plants (see summary in Fig. 2A). Because the combination of domains of WOX proteins differ among different species, we here classify *WOX* genes mainly based on their homeodomain (HD).

The genomes of the chlorophytes *Ostreococcus lucimarinus* and *Ostreococcus tauri* (Derelle et al. 2006; Palenik et al. 2007), the charophyte *Spirogloea muscicola* (Cheng et al. 2019), and the bryophytes *Physcomitrella patens* (Lang et al. 2018) and *Marchantia polymorpha* (Bowman et al. 2017) encode only AC-WOX proteins with the AC-HD and NTDW (Fig. 2A, B, E).

The genome of the lycophyte *Selaginella kraussiana* (Ge et al. 2016) contains a typical AC-*WOX* gene *SkWOX13d* encoding a protein with the NTDW and AC-HD, and some AC-WOX genes (*SkWOX13a*, *SkWOX13c*, and *SkWOX13e*) encoding proteins with only the AC-HD (Fig. 2A, B, E). Interestingly, *SkWOX13b* encodes a chimeric protein with a combination of the NTDW, AC-HD, and CTDW (Fig. 2A-C, E); and *SkWOX11c* encodes a chimeric protein with the NTDW and IC-HD (Fig. 2A, B, F). Both *SkWOX11a* and *SkWOX11b* encode proteins containing only a HD with a YYWFQNR (or YYWFNKR) sequence that appears to be transitional between the ancient (YNWFQNR) and the intermediate (FYWFQNR) clades (Fig. 2A, H). Therefore, IC-*WOX* is only partially established and WC-*WOX* is not present in the lycophyte *S. kraussiana*.

In the fern *Ceratopteris richardii* (Nardmann and Werr 2012), the AC-*WOX* gene *CrWOX13b* encodes a protein with the AC-HD (Fig. 2A, E), and the IC-*WOX* genes *CrWOXa* and *CrWOXb* encode IC-HD (Fig. 2A, F). The *CrWOX13a* gene encodes a chimeric protein with the NTDW, AC-HD, and CTDW (Fig. 2A-C, E). The WC-*WOX* gene *CrWUL* encodes a protein with the WC-HD and WUS box (Fig. 2A, D, G).

It seems that the NTDW, which is closely associated with the AC-HD, is notably ancient and might be present in the AC-WOXs in most green plants. The CTDW seems to have arisen in the common ancestor of vascular plants (lycophytes, ferns, and seed plants) associated with AC-WOX proteins. After the appearance of seed plants, the CTDW became associated with the IC-HD, thereby forming the typical IC-WOX proteins in seed plants. The WUS box probably arose in the common ancestor of ferns and seed plants, and is closely associated with the WC-HD in WC-WOX proteins.

Overall, the typical AC-WOX protein structure with the combination of the NTDW and AC-HD is an ancient structure in green plants. The typical IC-WOX protein structure with the combination of the IC-HD and CTDW is only present in seed plants, although IC-HD and CTDW are separately present in lycophytes and ferns. The typical WC-WOX protein structure with the combination of the WC-HD and WUS box is present in ferns and seed plants.

Although the IC-*WOX* gene *CrWOXa* in the fern *C. richardii* encodes a protein with the IC-HD and not the CTDW, it also plays a role in the establishment of the root founder cell (also known as the root apical mother cell in ferns) (Fig. 1E). *CrWOXa* is specifically expressed in both adventitious and lateral root founder cells (Nardmann and Werr 2012; Yu et al. 2020). Auxin is the key hormone that induces the initiation of adventitious and lateral roots by directly activating *CrWOXa* expression via AuxREs in its promoter (Yu et al. 2020). Exogenous application of artificial auxin (e.g. 2,4-dichlorophenoxyacetic acid (2,4-D) or picloram) can induce ectopic *CrWOXa* expression and enhance rooting (Yu et al. 2020). The division of the root founder cell results in the establishment of the tetrahedral root apical cell with four division planes, giving rise to three proximal merophytes and a distal merophyte (Hou and Blancaflor 2009; Hou and Hill 2004, 2002). The distal merophyte serves as the root cap initial cell, and the proximal merophytes divide to form all the root cells except the root cap (Hou and Blancaflor 2009; Hou and Hill 2004, 2002). During the division of the root founder cell to form the root apical cell and merophytes, CrWOXa might activate the expression of the WC-*WOX* gene *CrWUL*, which is restricted to the proximal merophytes (Nardmann and Werr 2012; Yu et al. 2020). Therefore, the auxin-*CrWOXa*-*CrWUL* pathway in root initiation in *C. richardii* is similar to the auxin-*WOX11*-*WOX5* pathway in adventitious root initiation in *Arabidopsis*. It has been hypothesized that the IC-*WOX* gene may have been recruited in the root founder cell in the common ancestor of ferns and seed plants for auxin-induced root initiation (Yu et al. 2020).

## Conclusion and perspectives

In conclusion, *Arabidopsis WOX11* plays a key role in founder cells to initiate new organs during the regeneration of adventitious roots from detached leaves, the regeneration of adventitious lateral roots from wounded primary roots, and callus formation in tissue culture. The overall role of *WOX11* is to establish the founder cells guided by auxin, and promote the transition of founder cells into the root/callus primordium. However, many questions remain unanswered. For example, *WOX11* is a direct target of the auxin signaling pathway, but why does auxin activate *WOX11* in root/callus founder cells and not in other cell types (e.g. mesophyll cells)? Which ARF(s) is/are responsible for *WOX11* activation in root/callus founder cells? What is the molecular mechanism that ensures that *WOX11* is repressed in the root/callus primordium? How does *WOX11* cooperate with auxin and other gene networks to initiate the root/callus primordium? Is WOX11 involved in sub-cellular regulation of plant regeneration? *WOX11* is also expressed in the proto-xylem in the root tip (Liu et al. 2014b; Sheng et al. 2017), but what is its function in these cells? It is important to address all these questions to understand the role of *WOX11* in plant regeneration and other developmental processes.

The role of *WOX11* in regeneration in *Arabidopsis* might be inherited from its role in root founder cell establishment in the common ancestor of seed plants and ferns. Besides its role in regeneration, *WOX11* is also involved in a wide range of plant developmental processes. In rice, for example, it is involved in root cap development (Wang et al. 2014), regulation of above-ground tissues (Cheng et al. 2018), and regulation of tiller angle (Hu et al. 2020; Y. Li et al. 2020b; Zhang et al. 2018a). Further studies should explore the common and specific roles of *WOX11* in different development processes. Furthermore, it will be interesting to study the roles of *WOX* genes encoding proteins with the IC-HD or CTDW in lycophytes to discover their ancient roles in vascular plant evolution (Ge et al. 2016), and to compare the regenerative mechanism and stem cell activities in animals and plants (Serrano-Ron et al. 2021; Yan et al. 2020).

## Data Availability

Not applicable.

## Abbreviations

WOX: Wuschel-related homeobox
AuxREs: Auxin response elements
ARF: AUXIN RESPONSE FACTOR
WOXCEs: WOX-binding cis elements
LBD: Lateral organ boundaries domain
RAM: Root apical meristem
ATXR2: Arabidopsis Trithorax-related 2
IBA: Indole-3-butanoic acid
CIM: Callus-inducing medium
SIM: Shoot-inducing medium
RIM: Root-inducing medium
CaM–IQM: Calmodulin iq-motif containing protein
PLT: PLETHORA
SCR: Scarecrow
IAA: Indole-3-acetic acid inducible
QC: Quiescent center
AC-WOX: Ancient-clade WOX
IC-WOX: intermediate-clade WOX
WC-WOX: WUS-clade WOX
NTDW: N-terminal domain of WOX
AC-HD: AC-type homeodomain
CTDW: C-terminal domain of WOX
IC-HD: IC-type homeodomain
WC-HD: WC-type homeodomain
2,4-D: 2,4-dichlorophenoxyacetic acid
